# Convergent evolution of body color between sympatric freshwater fishes via different visual sensory evolution

**DOI:** 10.1002/ece3.5211

**Published:** 2019-04-26

**Authors:** Javier Montenegro, Koji Mochida, Kumi Matsui, Daniel F. Mokodongan, Bayu K. A. Sumarto, Sjamsu A. Lawelle, Andy B. Nofrianto, Renny K. Hadiaty, Kawilarang W. A. Masengi, Lengxob Yong, Nobuyuki Inomata, Takahiro Irie, Yasuyuki Hashiguchi, Yohey Terai, Jun Kitano, Kazunori Yamahira

**Affiliations:** ^1^ Tropical Biosphere Research Center University of the Ryukyus Okinawa Japan; ^2^ Department of Biology Keio University Yokohama Japan; ^3^ School of Veterinary Medicine Azabu University Sagamihara Japan; ^4^ Faculty of Fisheries and Marine Science Halu Oleo University Kendari Indonesia; ^5^ Research Center for Biology LIPI Cibinong Indonesia; ^6^ Faculty of Fisheries and Marine Science Sam Ratulangi University Manado Indonesia; ^7^ Ecological Genetics Laboratory National Institute of Genetics Mishima Japan; ^8^ Center for Ecology and Conservation, College of Life and Environmental Sciences University of Exeter Cornwall UK; ^9^ Department of Environmental Science Fukuoka Women's University Fukuoka Japan; ^10^ AORI University of Tokyo Kashiwa Japan; ^11^ Department of Biology Osaka Medical College Osaka Japan; ^12^ Department of Evolutionary Studies of Biosystems The Graduate University for Advanced Studies Hayama Japan

**Keywords:** *Nomorhamphus*, opsin, *Oryzias*, phylogenetic constraint, sensory drive

## Abstract

Although there are many examples of color evolution potentially driven by sensory drive, only few studies have examined whether distinct species inhabiting the same environments evolve similar body colors via shared sensory mechanisms. In this study, we tested whether two sympatric freshwater fish taxa, halfbeaks of the genus *Nomorhamphus* and ricefishes of the genus *Oryzias* in Sulawesi Island, converge in both body color and visual sensitivity. After reconstructing the phylogeny separately for *Nomorhamphus* and *Oryzias* using transcriptome‐wide sequences, we demonstrated positive correlations of body redness between these two taxa across environments, even after phylogenetic corrections, which support convergent evolution. However, substantial differences were observed in the expression profiles of opsin genes in the eyes between *Nomorhamphus* and *Oryzias*. Particularly, the expression levels of the long wavelength‐sensitive genes were negatively correlated between the taxa, indicating that they have different visual sensitivities despite living in similar light environments. Thus, the convergence of body colorations between these two freshwater fish taxa was not accompanied by convergence in opsin sensitivities. This system presents a case in which body color convergence can occur between sympatric species via different mechanisms.

## 1 INTRODUCTION

Phenotypic convergence across distinct taxa has long attracted the attention of evolutionary biologists (e.g., Schluter, Clifford, Nemethy, & McKinnon, 2004; Arendt & Reznick, 2008; Losos, 2011; Pearce, 2011). Darwin (1859) explained it as a result of adaptations to similar environmental conditions, wherein natural selection independently acts on two or more organisms in nearly the same manner. Further, the evolution of the same phenotypic traits in distinct species and/or taxa that live in sympatry has often been recognized as strong evidence for the role of natural selection in phenotypic evolution (Muschick, Indermaur, & Salzburger, 2012; Rosenblum, 2006).

Convergence of body coloration between sympatric species is widespread across the animal kingdom (Huheey, 1960; Plowright & Owen, 1980; Robertson et al., 2011; Rosenblum, 2006). One of the most well‐known cases of body color resemblance between sympatric species through independent evolution is cryptic or warning colors against common or similar types of predators (Armbruster & Page, 1996; Rosenblum, 2006; Vignieri, Larson, & Hoekstra, 2010). For example, three lizard species of different genera display convergent blanched cryptic coloration on gypsum dunes (Rosenblum, 2006). Another is the warning color convergence between the monarch butterfly and the viceroy butterfly that have independently evolved similar wing patterns in response to predation pressures (Ritland & Brower, 1991).

Convergent body coloration could evolve as a result of signal adaptation to conspecifics. Particularly, it is theoretically possible that convergent evolution of body coloration could be driven by the same sensory adaptation to a common light environment that independently evolved in each species (Dick, Hinh, Hayashi, & Reznick, 2018). Although there are many examples of color evolution potentially driven by sensory drive, that is, sensory systems adapt to environments that lead to the evolution of signals (Cummings & Endler, 2018 for review), only few studies have examined whether distinct species sharing the same habitats acquire the same body colors because of the same sensory drive process.

Here, we tested whether body color convergence between sympatric freshwater fish species evolves via shared sensory mechanisms. Freshwater environments substantially vary in light environments, specifically in light intensity and spectrum composition of wavelengths that vary according to water depth, transparency, density and size of suspended particles, and canopy coverage, etc. (Bowmaker et al., 1994; Lythgoe, 1988). Many studies have demonstrated that divergent visual adaptations to these varying lighting environments could drive divergence in body coloration as social signals in freshwater fishes (Boughman, 2001; Cole & Endler, 2015; Maan, Hofker, Alphen, & Seehausen, 2006; Seehausen, 2015; Seehausen et al., 2008). For example, in the cichlid fishes of Lake Victoria, the red‐shifted light conditions in deeper waters coincide with a change in allele frequency of the red‐sensitive opsin gene, as well as the abundance of red male body coloration, possibly increasing both visual performance and male conspicuousness (Seehausen et al., 2008). In spring‐fed ponds with blue‐rich light environments, red color signals can evolve to enhance the contrast against the background together with higher expression of blue‐sensitive opsins (Fuller, 2002; Fuller, Carleton, Fadool, Spady, & Travis, 2004). Therefore, if sympatric species resemble each other in body coloration, one possible mechanism is sensory drive in a shared light environment.

**Figure 1:**
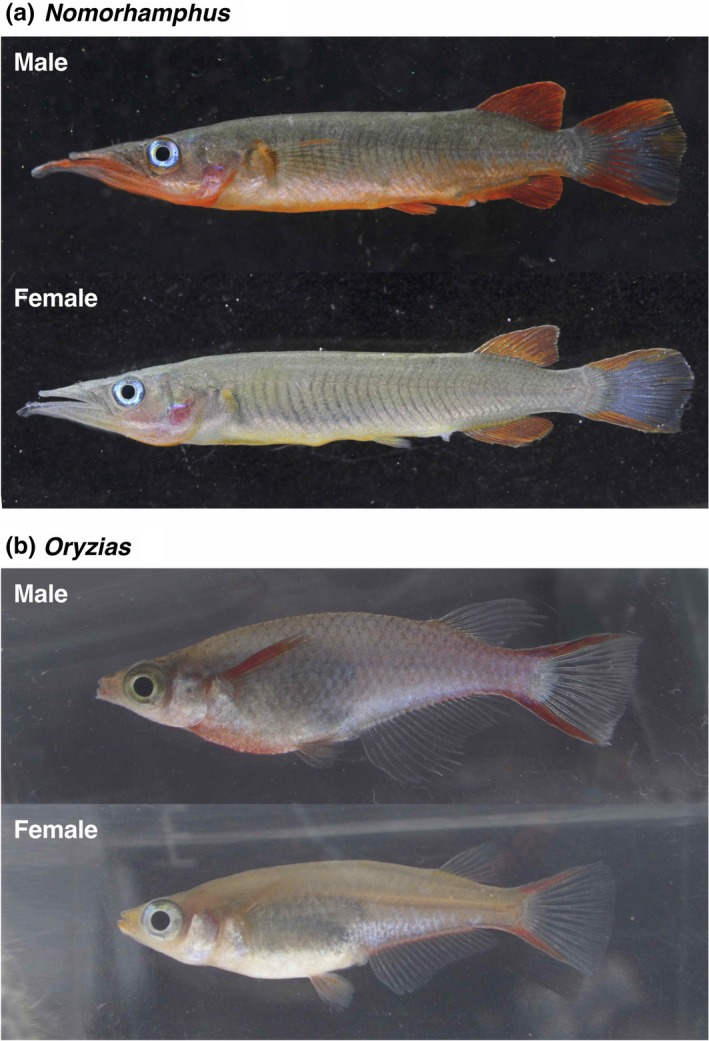
Representative photographs of (a) *Nomorhamphus* and (b) *Oryzias*

To test whether body color convergence can be driven by sensory adaptation to a common light environment, we focused on halfbeaks of the genus *Nomorhamphus* and ricefishes of the genus *Oryzias* in Sulawesi Island (Figure 1). These two genera belong to different families (Zenarchopteridae and Adrianichthyidae, respectively) and are highly diversified on this small island in the Indo‐Australian Archipelago (Huylebrouck, Hadiaty, & Herder, 2012, 2014; Meisner, 2001; Mokodongan & Yamahira, 2015). They co‐occur in a variety of habitats, ranging from a small muddy river to a big ancient lake with clear water (Kottelat, Whitten, Kartikasari, & Wirjoatmojo, 1993), providing a good model system to investigate the patterns of body color convergence in sympatry.

We specifically tested the hypothesis that both body color and visual tuning would show convergent evolution in the two genera. According to sensory drive hypothesis, two genera utilizing the same light environments may evolve similar visual sensitivity and mating signals. We first reconstructed the phylogeny separately for *Nomorhamphus* and *Oryzias* using transcriptome‐wide sequences and then demonstrated a positive correlation of body color between these two taxa, consistent with convergent evolution even after phylogenetic correction. Next, we tested whether the expression levels and amino acid sequences of opsin genes also show convergent evolution.

## 2 MATERIALS AND METHODS

### 2.1 Materials

The *Oryzias woworae* species group, composed of *Oryzias asinua* Parenti et al., 2013, *Oryzias wolasi* Prenti et al., 2013, and *O. woworae* Parenti & Hadiaty, 2010, is a monophyletic group of the family Adrianichthyidae endemic to southeastern Sulawesi, including Muna Island, a satellite island of Sulawesi (Mokodongan & Yamahira, 2015). The dorsal and ventral margins on the caudal fin are red to orange in both mature males and females, and the ventral area and pectoral fins are also red in some mature males (Figure 1a). Generally, body redness is more intense in males, suggesting that it may be a male mating signal in *Oryzias*.

The genus *Nomorhamphus* is a freshwater fish group of the family Zenarchopteridae endemic to Sulawesi and the Philippines (Huylebrouck, Hadiaty, & Herder, 2012, 2014; Meisner, 2001). During the course of this study, we found that the species *Nomorhamphus ebrardtii* (Popta, 1912) coexists with the *Oryzias* species throughout the geographic range of the *O. woworae* species group. They have characteristically yellowish‐to‐reddish fins and ventral areas (Figure 1b). Like in *Oryzias*, males have more intense red coloration, suggesting that this genus may also use the body color as a male mating signal.

*Oryzias* and *Nomorhamphus* share the same microhabitats in several localities in southeastern Sulawesi. In both *Oryzias* and *Nomorhamphus*, mature individuals are especially abundant near the shore, where males defend territories (K. Yamahira, personal observation). During mating of *Oryzias*, one male and one female align side‐by‐side, and the male wraps the female's body with his dorsal and anal fins. The pair then quivers for <30 min while the female externally releases her eggs, which are then fertilized by the male (B.K.A. Sumarto and A.B. Nofrianto, personal observation). On the other hand, *Nomorhamphus* reproduces by viviparity (Meisner, 2001). Males have an andropodium, which is used to pass a sperm bundle into female, and fertilization occurs internally (Meisner, 2001). However, the details of mating behaviors have not yet been reported.

### 2.2 Phylogenetic analyses

**Figure 2:**
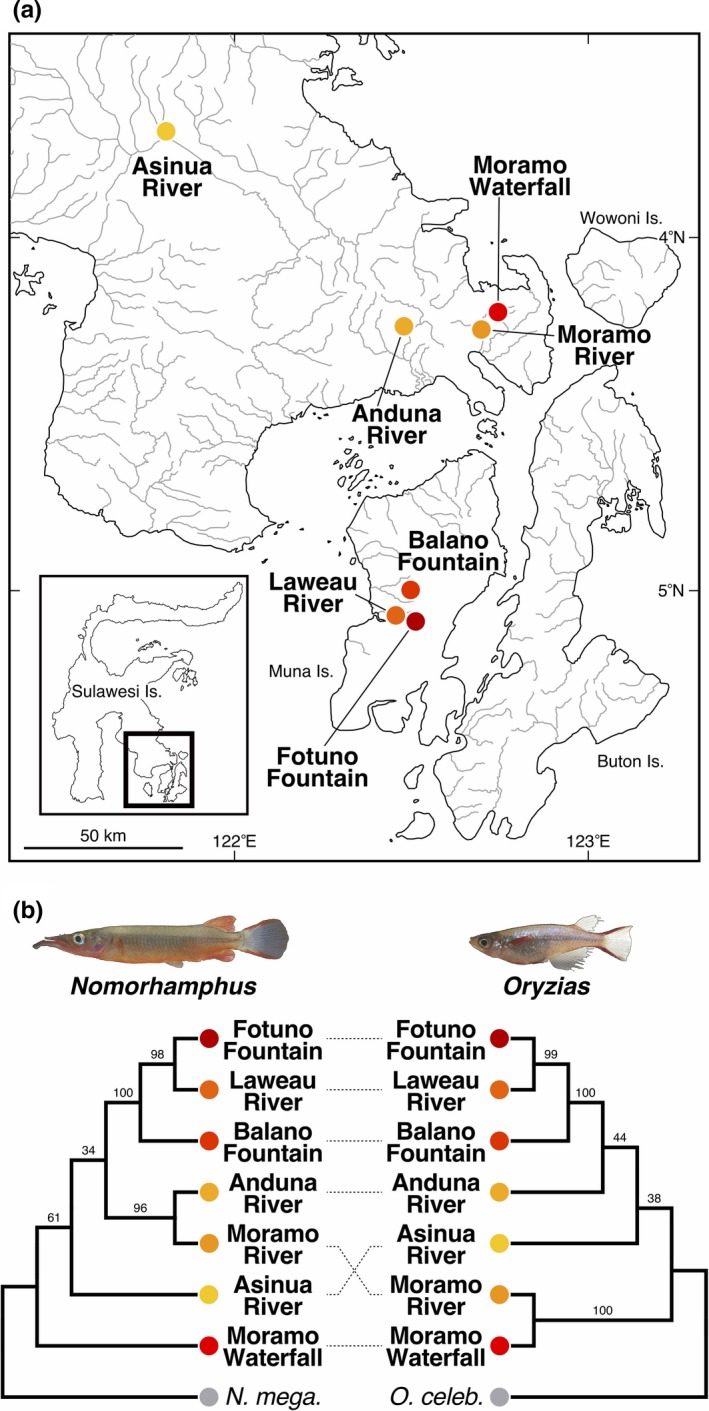
(a) Map showing the location of each of the seven collection sites and population trees of *Nomorhamphus* and *Oryzias*. (b) Population tree with biogeographic reconstruction of *Nomorhamphus* and *Oryzias* trees based on the 272 and 148 transcripts, respectively, using SVDquartets + PAUP*. Numbers on branches show bootstrap values

Three individuals (one adult male and two adult females) of each of the two genera, *Nomorhamphus* and *Oryzias*, were collected from seven localities (Asinua River, Anduna River, Moramo Waterfall, Moramo River, Fotuno Fountain, Laweau River, and Balano Fountain) throughout the southeastern arm of Sulawesi and on Muna Island (Figure 2a). A coalescent‐based population tree and a maximum likelihood (ML) phylogenetic tree were obtained separately for *Nomorhamphus* and *Oryzias* based on the RNA‐Seq data of an eye of each individual, using the methods described by Mokodongan et al. (2018) (Data S1 and Tables S1 and S2 for details). We also calculated the number of net nucleotide substitutions per site between the two populations (*d*_A_), using the transcriptome‐wide sequences (Data S1 for details). A population‐averaged phylogenetic tree was then reconstructed with the neighbor‐joining (NJ) method, using the matrix of *d*_A_ values for each pair among the seven populations. The *d*_A_ matrices (Table S3) were also used for the phylogenetic corrections below (see Statistical analyses below).

### 2.3 Measurement of body redness

Five adult males and five adult females of both *Nomorhamphus* and *Oryzias* were collected from each of the seven localities (except for Moramo Waterfall, where only four *Oryzias* females were collected) and brought back life to the indoor laboratory. After an acclimation period, they were put in a small acrylic tank and photographed from the side with two color charts (CasMatch, Funakoshi), using a digital camera (Stylus TG‐3, Olympus). All pictures were color‐corrected using the color charts according to the manufacturer's instructions, and the body area of each individual, including fins, was clipped out from their pictures, using Photoshop CC (Adobe). First, we measured the ratio of the red areas to the total body area as an index of body redness. Areas were selected and quantified as the number of pixels, using the software ImageJ (Abràmoff, Magalhães, & Ram, 2004). Red areas were manually selected with an HSB (hue, saturation, and brightness) threshold of *H* = 245–15 (345.9°–21.2°), *S* = 60–255 (23.5%–100%), and *B* = 160–255 (62.7%–100%), which was obtained using the “True Red” squares on the color charts of all analyzed pictures. Second, we also quantified an opponent color measure of redness versus greenness (R/G), as another index of body redness, using a RGB color space (R/G = [R – G]/[R + G]). The mean R/G was obtained from the red areas selected based on the HSB threshold above. The first and second indices are expected to represent the distribution and density of erythrophores and/or xanthophores, respectively. To reduce possible effects of the camera sensors’ spectral sensitivity and camera's nonlinear processing on the redness assessment (Stevens, Parraga, Cuthill, Partridge, & Troscianko, 2007), all pictures were taken using a single camera under standardized lighting.

To demonstrate that the among‐population variation in body redness persists even in a laboratory common environment, we measured the red area relative to the total body area of laboratory‐raised individuals of *Nomorhamphus* and *Oryzias* originating from the Fotuno Fountain and Asinua River as described above (Data S1 for details). Additionally, the carotenoid concentration (nmol/g) was measured for wild individuals collected from the Fotuno Fountain and Asinua River locations (Data S1 for details).

### 2.4 Expression levels and amino acid sequences of opsin genes

Variations in opsin expression levels have been often investigated as a proxy for variations in color sensitivity in several organisms, including fishes (e.g., Carleton & Kocher, 2001; Fuller et al., 2004; Sakai, Kawamura, & Kawata, 2018). Here, we investigated the opsin expression levels of the seven *Nomorhamphus* and seven *Oryzias* populations, using the RNA‐Seq data of each of the three above‐mentioned individuals. The raw sequence reads were first trimmed using CLC Genomics Workbench 8.0 (CLC bio) as previously described by Ishikawa et al. (2017): the nucleotides with low‐quality scores (<20) and two or more ambiguous nucleotides at the ends were removed. The trimmed sequence reads were then mapped to the opsin cDNA sequences (SWS1, SWS2a, SWS2b, RH2a, RH2b, RH2c, LWSa, and LWSb) annotated in the Ensembl medaka reference genome (MEDAKA1 Version 74.1) using the RNA‐Seq analysis software included in the CLC Genomics Workbench to calculate the reads per kilobase of exon per million mapped reads (RPKM) with the following parameters: maximum number of mismatches allowed = 2; minimum length fraction = 0.9; minimum similarity fraction = 0.8; unspecific match limit = 10. Due to high sequence similarity, we combined the RPKM values of SWS2a and SWS2b (SWS2a + 2b), LWSa and LWSb (LWSa + b), and RH2b and RH2c (RH2b + 2c).

Furthermore, we obtained the consensus sequence from the mapped data (see above) for each individual. These consensus sequences were translated to amino acid sequences and aligned with the amino acid sequence of bovine RH1 (GenBank accession number: NP_001014890.1) using ClustalW in MEGA ver. 6 (Tamura, Stecher, Peterson, Filipski, & Kumar, 2013). The spectrum tuning sites were predicted for each opsin according to the methods described by Yokoyama, Yang, and Starmer (2008) and Janz and Farrens (2001).

### 2.5 Statistical analyses

Among‐population and sex differences in the relative body red area of wild‐caught individuals were tested separately for each genus by applying the analysis of variance (ANOVA) to the natural logarithm (ln) of the original data. The population, sex, and their interactions were incorporated into the model as fixed‐effect factors. The same ANOVA was also performed on the mean R/G. Similarly, among‐population differences in the relative expression levels of LWSa and LWSb (LWSa + b) were tested separately for each genus by applying one‐way ANOVA to the ln‐transformed data with the sexes pooled (because of small sample size). These analyses were conducted using JMP statistical software (ver. 13.2.0 for Mac, SAS Institute).

We also tested whether the relative body red area in wild individuals showed a similar among‐population pattern between the two genera. Pearson's correlation coefficients (*r*s) in the population‐mean red area between *Oryzias* and *Nomorhamphus* were calculated separately for males and females. For testing the null hypothesis of *r* = 0, the degree of freedom could be overestimated by referring to Student's *t* distribution because the mean red area was not statistically independent among populations within the same genus, that is, the phylogenetically close populations might be more similar in redness because of “phylogenetic constraint” (Felsenstein, 1985; Harvey & Pagel, 1991). We, instead, implemented a parametric bootstrap method (Efron & Tibshirani, 1993) to generate the reference distribution of *r* for calculating the *p*‐value, which takes into account the phylogenetic relationship among the populations derived from the above‐mentioned genetic distance (i.e., *d*_A_) matrices (Appendix S1). Similarly, the correlation in the population‐mean R/G between *Oryzias* and *Nomorhamphus* was tested separately for males and females using the same methods (Appendix S1). We also tested whether the two genera showed a similar among‐population pattern in opsin gene expression by a correlation test with phylogeny into account. Here, we used population‐averaged expression levels and tested each opsin gene separately (Appendix S1). Pearson's correlation coefficients between the population‐mean red area in males and the population‐averaged LWS expression level were also calculated separately for *Nomorhamphus* and *Oryzias*.

## 3 RESULTS

### 3.1 Phylogeny of *Nomorhamphus* and *Oryzias*

The coalescent‐based population tree of *Nomorhamphus* based on the RNA‐Seq data revealed that the populations in Muna Island (i.e., Fotuno Fountain, Laweau River, and Balano Fountain) were monophyletic (Figure 2b). The population tree of *Oryzias* yielded essentially the same topology as that of *Nomorhamphus* (Figure 2b), although several topological differences were also evident between the two genera on the side of the Sulawesi mainland. The ML phylogenetic tree and the NJ tree based on the *d*_A_ values were also similar between *Nomorhamphus* and *Oryzias* (Figure S1).

### 3.2 Parallelism in body redness between *Nomorhamphus* and *Oryzias*

**Figure 3:**
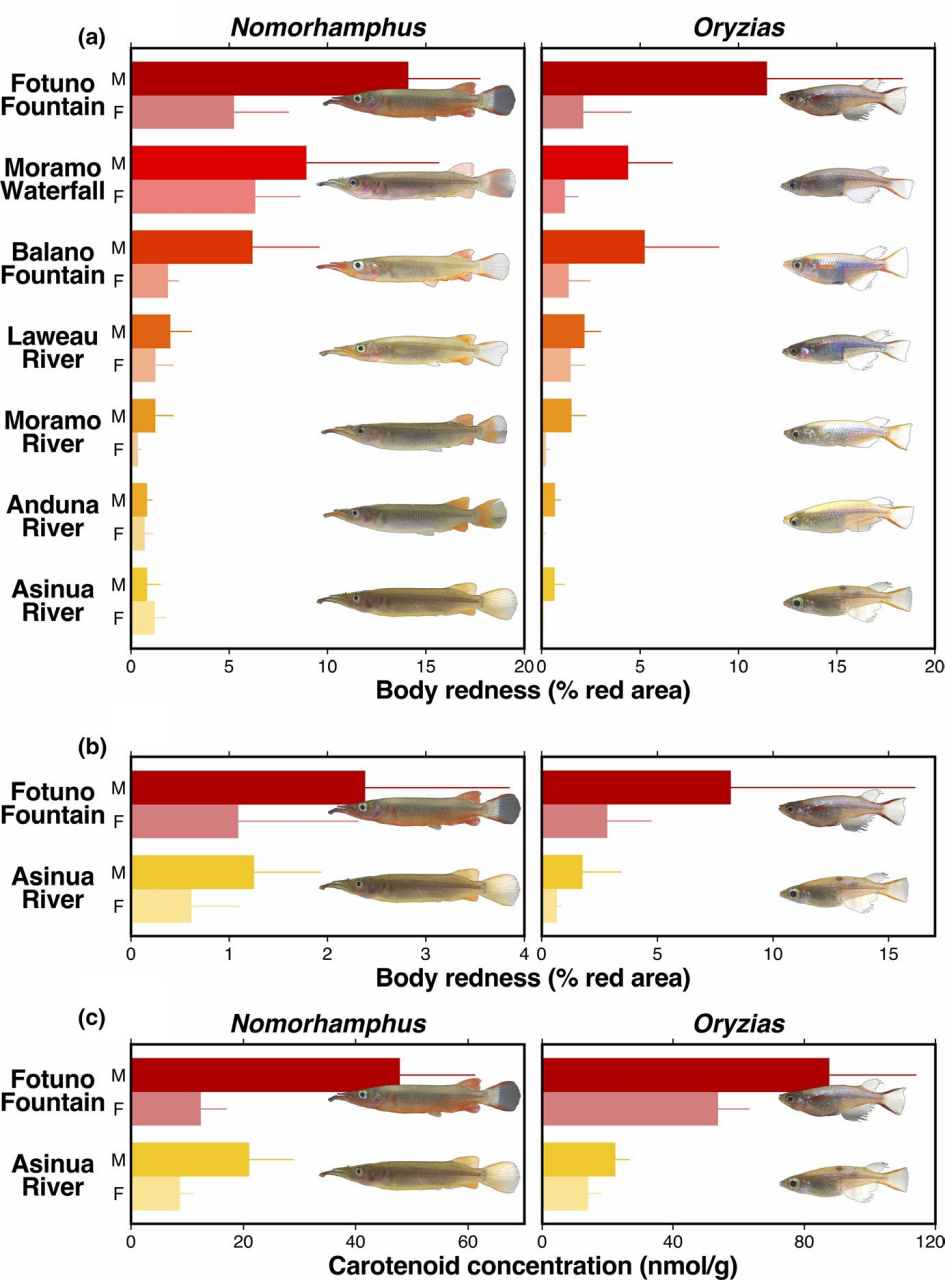
Body redness measured as the ratio of the red areas to the total body area of (a) the wild individuals and (b) the laboratory‐raised individuals, and (c) carotenoids concentration of the wild individuals of *Nomorhamphus* and *Oryzias*. Mean redness (±*SD*) is shown separately for males and females. Images of fishes are those of males

Substantial variations in body redness were observed among populations in both genera when measured as the ratio of the red areas to the total body area (Figure 3a). In *Nomorhamphus*, there was a significant difference in body redness among populations (*p* < 0.0001), although the interaction between population and sex was also marginally significant (*p* = 0.0496). *Oryzias* also significantly differed in mean redness among populations (*p* < 0.0001), while the population‐by‐sex interaction was not significant in this genus (*p* = 0.1498). In both genera, males were redder than females (Figure 3a; *Nomorhamphus*: *p* = 0.0453; *Oryzias*: *p* < 0.0001). Body redness measured as the mean R/G also significantly varied among populations in both genera (Figure S2; *Nomorhamphus*: *p* = 0.0004; *Oryzias*: *p* < 0.0001).

The population‐mean red area relative to the total body area was positively correlated between *Nomorhamphus* and *Oryzias* both in males (*r* = 0.9598, *N* = 7) and females (*r* = 0.6479, *N* = 7). After controlling for the positive autocorrelation due to phylogenetic relationships (Appendix S1), the observed correlation coefficient was statistically significant in males (*p* = 0.0019, *e* ^−0.1^ ≤ *ρ* ≤ 1; Table Appendix S1.1), but not in females (*p* = 0.1146, *e* ^−0.1^ ≤ *ρ* ≤ 1; Table Appendix S1.1). On the other hand, the correlation in the population‐mean R/G was significant neither in males (*r* = 0.1436, *N* = 7, *p* = 0.4099, *e* ^−0.1^ ≤ *ρ* ≤ 1; Table Appendix S1.2) nor females (*r* = 0.1639, *N* = 7, *p* = 0.3985, *e* ^−0.1^ ≤ *ρ* ≤ 1; Table Appendix S1.2).

Comparisons of the laboratory‐raised individuals revealed that the Fotuno Fountain individuals had larger red area relative to the body area than the Asinua River individuals in both *Nomorhamphus* and *Oryzias* (Figure 3b), which is consistent with the observed difference observed in the wild‐caught fish. In both populations of both genera, the laboratory‐raised males had larger red patches than the laboratory‐raised females.

Analyses of the carotenoid concentration revealed that the Fotuno Fountain individuals accumulated more carotenoids than the Asinua River individuals both in *Nomorhamphus* and *Oryzias* (Figure 3c). In both populations of both genera, the carotenoids were more accumulated in males than in females.

### 3.3 Expression levels and amino acid sequences of opsin genes

**Figure 4:**
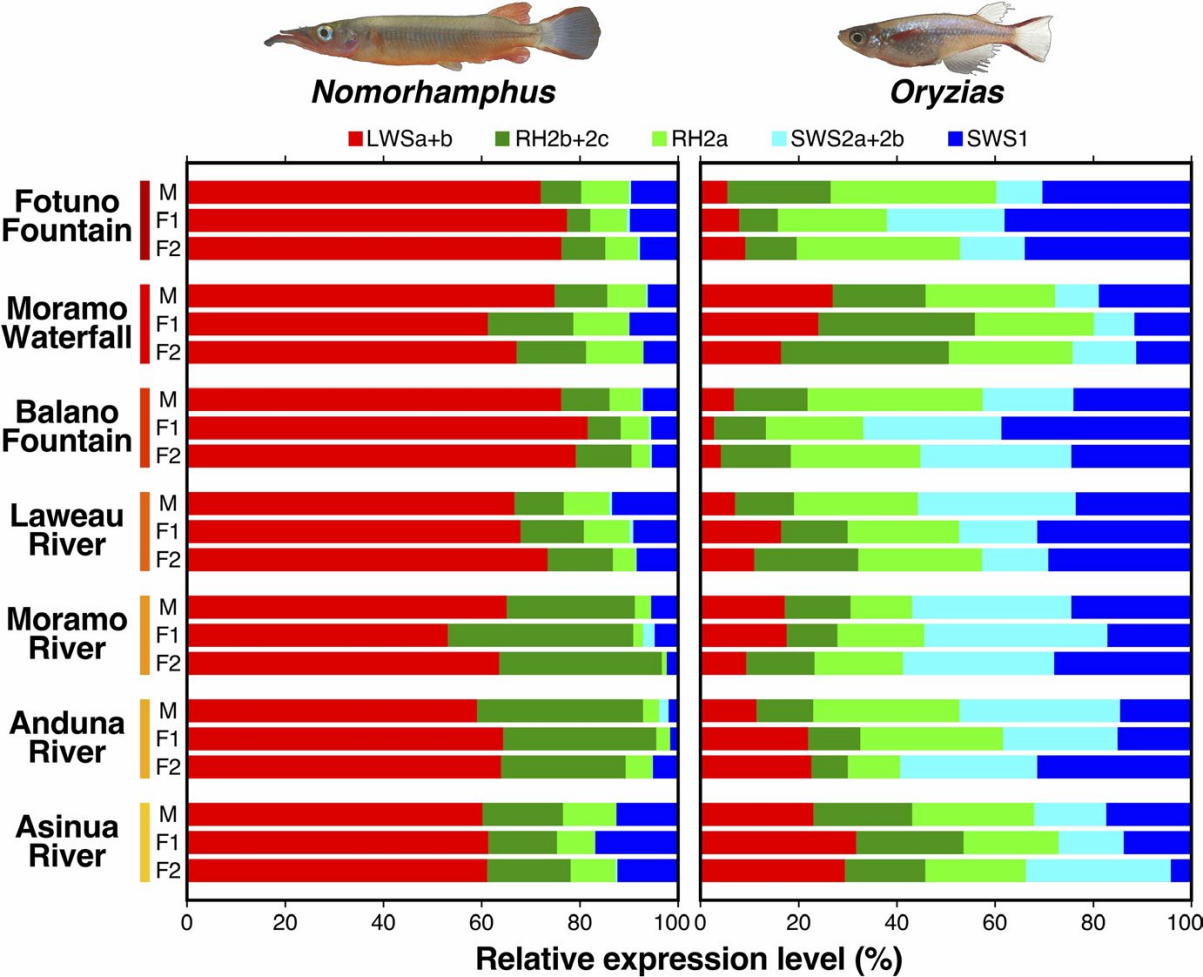
Relative mRNA expression levels (relative reads per kilobase of exon per million mapped reads [RPKM] based on the RNA‐Seq data) of the opsin genes of each wild individual collected from the seven local populations of *Nomorhamphus* and *Oryzias*

Overall patterns of variation in opsin gene expression seemed opposite between *Nomorhamphus* and *Oryzias* (Figure 4). In *Nomorhamphus*, the redder populations, such as the Fotuno Fountain and Balano Fountain populations, showed higher LWS opsin expression, whereas the relatively less red populations, such as the Asinua River and Anduna River populations, showed lower LWS expression. In *Oryzias*, however, the redder populations, such as the Fotuno Fountain population, showed rather lower LWS expression, while the less red populations, such as the Asinua River, showed higher LWS expression. Both in *Nomorhamphus* and *Oryzias*, the mean expression level of the LWS opsin genes (LWSa + b) was significantly different among the local populations (*Nomorhamphus*: *p* = 0.0008; *Oryzias*: *p* < 0.0001). The correlation in the mean LWS expression between the two genera was negative across the seven populations (*r* = –0.7956, *N* = 7). After correcting for phylogenetic relationships (Appendix S1), the observed correlation coefficient remained statistically significant (*p* = 0.0463, *e* ^−0.1^ ≤ *ρ* ≤ 1; Table Appendix S1.3). The LWS expression level showed a trend of positive and negative correlations with the relative body red area in males in *Nomorhamphus* (*r* = 0.6969, *N* = 7) and *Oryzias* (*r* = –0.5939, *N* = 7), respectively, although the correlations were not significant (*Nomorhamphus*: *p* = 0.0818; *Oryzias*: *p* = 0.1597).

On the other hand, the correlation coefficients for all other opsin genes were weak and nonsignificant (RH2b + 2c, *r* = –0.3324; RH2a, *r* = 0.5095; SWS1, *r* = –0.2120; *N* = 7, *p* > 0.05, *e* ^−0.1^ ≤ *ρ* ≤ 1 for all cases; Table Appendix S1.3), except for SWS2a + 2b, which showed a significantly positive correlation when weak phylogenetic effects were assumed (*r* = 0.7764, *N* = 7, *p* = 0.0491, *e* ^−1^ ≤ *ρ* ≤ 1; Table Appendix S1.3). Overall, LWS was expressed at higher levels in *Nomorhamphus* than in *Oryzias*; the *F* test applied on the full model revealed that the effect of genus was significant (*p* < 0.0001).

The amino acid sequence comparison of each opsin gene indicated that the spectral sensitivities of the opsin pigments were predicted not to differ substantially among the local populations in any opsin genes both in *Nomorhamphus* and *Oryzias* (Figure S3). The spectral sensitivities of SWS1, SWS2b, RH2b, and RH2c pigments were predicted not to differ even between *Nomorhamphus* and *Oryzias*. One amino acid replacement was found at the spectrum tuning site of RH2a (position 292 in the bovine RH1) between the two genera; the replacement of alanine in *Nomorhamphus* with serine in *Oryzias* at that site was predicted to result in approximately 10‐nm blue shift of the absorption spectra in RH2a pigments (Yokoyama et al., 2008). A replacement of tyrosine in *Oryzias* with phenylalanine in *Nomorhamphus* was also found at the spectrum tuning site of LWSa and LWSb (corresponding to the position 178 in the bovine RH1); this substitution was predicted to result in approximately 18‐nm blue shift of the absorption spectra in LWSa pigments (Janz & Farrens, 2001).

## 4 DISCUSSION

**Figure 5:**
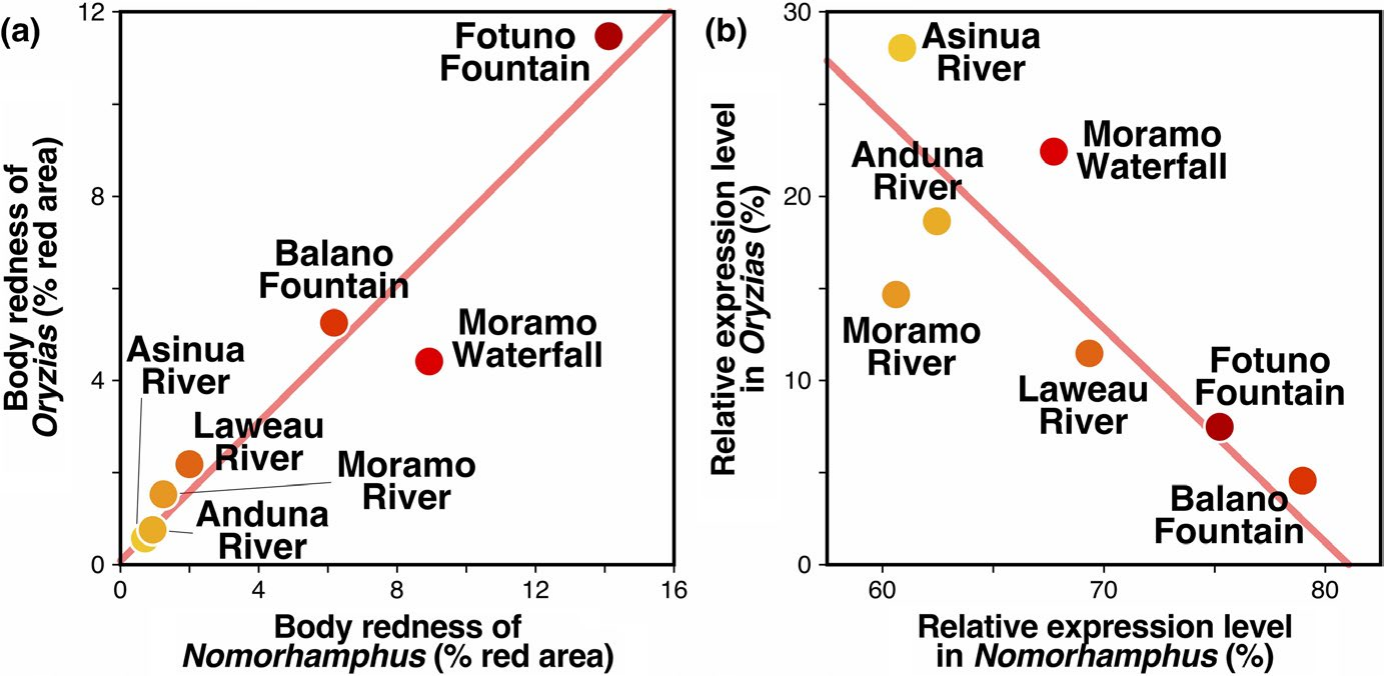
Correlation of (a) the population‐mean male redness measured as the ratio of the red areas to the total body area and (b) the population‐mean expression level of the LWS opsin genes (LWSa + b) between *Nomorhamphus* and *Oryzias*. The regression line in each plot represents a reduced‐major axis regression

Our analyses of body redness revealed a strong positive correlation in body coloration between *Nomorhamphus* and *Oryzias* across the studied environments (Figure 5a). In habitats where *Oryzias* were redder, *Nomorhamphus* also tended to be more reddish, and vice versa. Furthermore, we demonstrated that the among‐population variation in the body redness of *Nomorhamphus* and *Oryzias* persisted even in a laboratory common environment, indicating that the variation in coloration has a genetic basis. Moreover, we found that the among‐population variation in red coloration was associated with the carotenoid concentration in both genera, suggesting that they use the same resources for body coloration. Our case presents a clear example of body color convergence between sympatric taxa, offering great opportunities to further investigate the mechanisms of body color convergence.

One of the hypotheses for convergence in body color was sensory drive that occurs independently but similarly in the two taxa sharing the same light environments. According to the sensory drive hypothesis, two genera utilizing the same light environments are predicted to evolve both similar visual tuning and mating signals. The significantly male‐biased body redness supports the view that the red coloration may be used as a male mating signal in both genera. However, substantial differences were observed in the expression profiles of opsin genes between *Nomorhamphus* and *Oryzias*. Particularly, the expression levels of the LWS genes were negatively correlated between the taxa across environments (Figure 5b). As a result, we found that the LWS expression level was positively and negatively correlated with the male body redness across populations in *Nomorhamphus* (Figure S4a) and in *Oryzias* (Figure S4b), respectively. Moreover, the two genera differed in the overall expression levels of the LWS genes; the *Nomorhamphus* individuals exhibited much higher expression than the *Oryzias* individuals, indicating that they have different visual senses even under common light environments. While opsin expression is known to be plastic in response to lighting environments (Nandamuri, Yourick, & Carleton, 2017), these findings indicate that the convergence of body colorations between these two sympatric freshwater fish taxa is not accompanied by convergence in visual sensitivity.

How does body coloration converge without visual sense convergence then? One possible explanation is that sensory drive acts in both genera, but differently. It should be noted that the coevolution pattern between body color and visual sense in *Oryzias* might be similar to the case of the bluefin killifish *Lucania goodei* (Fuller, 2002; Fuller, Fleishman, Leal, Travisx, & Loew, 2003). During the course of field collections, we observed under water that long wavelengths are apparently less dominant in some habitats, such as in the fountain habitats where fishes are reddish, than in the riverine habitats where fishes are less reddish (Figure S5). The among‐population variation in the expression levels of the LWS opsin genes in *Oryzias* might reflect visual adaptations to local light environments; they might exhibit higher sensitivity to dominant wavelengths in each habitat. If this is the case, the reddish male body in the fountain‐inhabiting *Oryzias* might have evolved to maximize the contrast between the signalers and the background. Such a high‐contrast male body coloration has been suggested to be driven by sensory drive in the bluefin killifish (Fuller, 2002; Fuller et al., 2003). On the other hand, a redder body might have not evolved in the *Oryzias* males of the riverine habitats where body redness does not increase the contrast to the background. However, this scenario was not applicable to the *Nomorhamphus* individuals, which exhibited an opposite pattern in the expression profiles of opsin genes.

On the other hand, the coevolution pattern between body color and visual sense in *Nomorhamphus* might be similar to that reported in lake sticklebacks, in which female perceptual sensitivity to red light increases with decreasing extent of long wavelengths in habitat environments, and the male body redness is tuned to the female perceptual sensitivity (Boughman, 2001; but see Rennison, Owens, Heckman, Schluter, & Veen, 2016). Boughman (2001) argued that this positive covariance could be caused by the male signals evolving to match a pre‐existing bias. However, it is unclear how the visual bias to redness pre‐existed in the habitats where long wavelengths were less dominant. Because we observed that the *Nomorhamphus* individuals prey on the *Oryzias* individuals in aquariums, the visual sensitivity of the former might have been tuned to facilitate the detection of *Oryzias* as prey. If so, the among‐population variation in the male redness of *Nomorhamphus* might have been driven by the pre‐existing variation in the visual sensitivity that reflects the variation in the body redness of *Oryzias*. Further studies on the trophic ecology of these two species and habitat light environments will help to understand why *Nomorhamphus* and *Oryzias* have different visual senses even under common lighting environments.

In summary, our results revealed that the convergence of body coloration between *Nomorhamphus* and *Oryzias* was not accompanied by convergence in visual sensitivity. We propose that the convergence of body redness was driven by taxon‐specific selection mechanisms. It is often assumed that the phenotypic convergence between distinct sympatric taxa reflects independent evolution resulting from being exposed to a common selection pressure. However, our *Nomorhamphus*–*Oryzias* system presents a case that sympatric taxa converge in color likely by different mechanisms.

## CONFLICT OF INTEREST

None declared.

## AUTHOR CONTRIBUTIONS

Montenegro, Mochida, Mokodongan, Sumarto, Lawelle, Nofrianto, Hadiaty, Masengi, Kitano, and Yamahira conducted fieldwork. Montenegro, Mochida, Matsui, Sumarto, Inomata, Kitano, and Yamahira performed laboratory work, and Montenegro, Yong, Irie, Hashiguchi, Terai, and Yamahira conducted the analyses. Montenegro, Yong, Irie, Hashiguchi, Terai, Kitano, and Yamahira wrote the manuscript. All authors read and approved the final manuscript.

## DATA AVAILABILITY STATEMENT

All raw data were deposited in the DNA Data Bank of Japan (DDBJ) Sequence Read Archive (SRA) under the accession number DRA006423, and the concatenated contig alignments and their partition information are archived on Dryad (https://doi.org/10.5061/dryad.21705km).

## References

[ece35211-bib-0001] Abràmoff, M. D. , Magalhães, P. J. , & Ram, S. J. (2004). Image processing with ImageJ. Biophotonics International, 11, 36–42.

[ece35211-bib-0002] Arendt, J. , & Reznick, D. (2008). Convergence and parallelism reconsidered: What have we learned about the genetics of adaptation? Trends in Ecology & Evolution, 23, 26–32. 10.1016/j.tree.2007.09.011 18022278

[ece35211-bib-0003] Armbruster, J. W. , & Page, L. M. (1996). Convergence of a cryptic saddle pattern in benthic freshwater fishes. Environmental Biology of Fishes, 45, 249–257. 10.1007/bf00003092

[ece35211-bib-0004] Boughman, J. W. (2001). Divergent sexual selection enhances reproductive isolation in sticklebacks. Nature, 411, 944–948. 10.1038/35082064 11418857

[ece35211-bib-0005] Bowmaker, J. K. , Govardovskii, V. I. , Shukolyukov, S. A. , Zueva, L. V. J. , Hunt, D. M. , Sideleva, V. G. , & Smirnova, O. G. (1994). Visual pigments and the photic environment: The cottoid fish of Lake Baikal. Vision Research, 34, 591–605. 10.1016/0042-6989(94)90015-9 8160379

[ece35211-bib-0006] Carleton, K. L. , & Kocher, T. D. (2001). Cone opsin genes of african cichlid fishes: Tuning spectral sensitivity by differential gene expression. Molecular Biology and Evolution, 18, 1540–1550. 10.1093/oxfordjournals.molbev.a003940 11470845

[ece35211-bib-0007] Cole, G. L. , & Endler, J. A. (2015). Variable environmental effects on a multicomponent sexually selected trait. American Naturalist, 185, 452–468. 10.1086/680022 25811082

[ece35211-bib-0008] Cummings, M. E. , & Endler, J. A. (2018). 25 Years of sensory drive: The evidence and its watery bias. Current Zoology, 64, 471–484. 10.1093/cz/zoy043 30108628 PMC6084598

[ece35211-bib-0009] Darwin, C. (1859). On the origin of species by means of natural selection, or the preservation of favoured races in the struggle for life. London, UK: John Murray. PMC5184128 30164232

[ece35211-bib-0010] Dick, C. , Hinh, J. , Hayashi, C. Y. , & Reznick, D. N. (2018). Convergent evolution of coloration in experimental introductions of the guppy (*Poecilia reticulata*). Ecology and Evolution, 8, 8999–9006. 10.1002/ece3.4418 30271561 PMC6157698

[ece35211-bib-0011] Efron, B. , & Tibshirani, R. J. (1993). An Introduction to the Bootstrap. London, UK: Chapman & Hall/CRC.

[ece35211-bib-0012] Felsenstein, J. (1985). Phylogenies and the comparative method. American Naturalist, 125, 6389–15. 10.1086/284325

[ece35211-bib-0013] Fuller, R. C. (2002). Lighting environment predicts relative abundance of male colour morphs in bluefin killifish (*Lucania goodei*) populations. Proceedings of the Royal Society of London Series B, 269, 1457–1465. 10.1098/rspb.2002.2042 12137575 PMC1691049

[ece35211-bib-0014] Fuller, R. C. , Carleton, K. L. , Fadool, J. M. , Spady, T. C. , & Travis, J. (2004). Population variation in opsin expression in the bluefin killifish, *Lucania goodei*: A real‐time PCR study. Journal of Comparative Physiology A, 190, 147–154. 10.1007/s00359-003-0478-z 14685760

[ece35211-bib-0015] Fuller, R. C. , Fleishman, L. J. , Leal, M. , Travisx, J. , & Loew, E. (2003). Intraspecific variation in retinal cone distribution in the bluefin killifish, *Lucania * *Goodei* . Journal of Comparative Physiology A, 189, 609–616. 10.1007/s00359-003-0435-x 12879350

[ece35211-bib-0016] Harvey, P. H. , & Pagel, M. D. (1991). The comparative method in evolutionary biology. Oxford, UK: Oxford Univ. Press.

[ece35211-bib-0017] Huheey, J. E. (1960). Mimicry in the color pattern of certain Appalachian salamanders. Journal of the Elisha Mitchell Scientific Society, 76, 246–251.

[ece35211-bib-0018] Huylebrouck, J. , Hadiaty, R. K. , & Herder, F. (2012). *Nomorhamphus rex*, a new species of viviparous halfbeak (Atherinomorpha: Beloniformes: Zenarchopteridae) endemic to Sulawesi Selatan, Indonesia. Raffles Bulletin of Zoology, 60, 477–485.

[ece35211-bib-0019] Huylebrouck, J. , Hadiaty, R. K. , & Herder, F. (2014). Two new species of viviparous halfbeaks (Atherinomorpha: Beloniformes: Zenarchopteridae) endemic to Sulawesi Tenggara, Indonesia. Raffles Bulletin of Zoology, 62, 200–209.

[ece35211-bib-0020] Ishikawa, A. , Kusakabe, M. , Yoshida, K. , Ravinet, M. , Makino, T. , Toyoda, A. , … Kitano, J. (2017). Different contributions of local‐and distant‐regulatory changes to transcriptome divergence between stickleback ecotypes. Evolution (N.Y.), 71, 565–581. 10.1111/evo.13175 28075479

[ece35211-bib-0021] Janz, J. M. , & Farrens, D. L. (2001). Engineering a functional blue‐wavelength‐shifted rhodopsin mutant. Biochemistry, 40, 7219–7227. 11401569 10.1021/bi002937i

[ece35211-bib-0022] Kottelat, M. , Whitten, A. J. , Kartikasari, S. N. , & Wirjoatmojo, S. (1993). Freshwater fishes of Western Indonesia and Sulawesi. Hong Kong, China: Periplus Editions.

[ece35211-bib-0023] Losos, J. B. (2011). Convergence, adaptation, and constraint. Evolution (N.Y.), 65, 1827–1840. 10.1111/j.1558-5646.2011.01289.x 21729041

[ece35211-bib-0024] Lythgoe, J. N. (1988). Light and vision in the aquatic environment In Atema J., Fay R. R., Popper A. N., & Tavolga W. N. (Eds). Sensory biology of aquatic animals (pp. 57–82). New York, NY: Springer.

[ece35211-bib-0025] Maan, M. E. , Hofker, K. D. , van Alphen, J. J. , & Seehausen, O. (2006). Sensory drive in cichlid speciation. American Naturalist, 167, 947–954. 10.2307/3844750 16615032

[ece35211-bib-0026] Meisner, A. D. (2001). Phylogenetic systematics of the viviparous halfbeak genera *Dermogenys* and *Nomorhamphus* (Teleostei: Hemiramphidae: Zenarchopterinae). Zoological Journal of the Linnean Society, 133, 199–283. 10.1111/j.1096-3642.2001.tb00690.x

[ece35211-bib-0027] Mokodongan, D. F. , Montenegro, J. , Mochida, K. , Fujimoto, S. , Ishikawa, A. , Kakioka, R. , … Yamahira, K. (2018). Phylogenomics reveals habitat‐associated body shape divergence in *Oryzias woworae* species group (Teleostei: Adrianichthyidae). Molecular Phylogenetics and Evolution, 118, 194–203. 10.1016/j.ympev.2017.10.005 29024751

[ece35211-bib-0028] Mokodongan, D. F. , & Yamahira, K. (2015). Origin and intra‐island diversification of Sulawesi endemic Adrianichthyidae. Molecular Phylogenetics and Evolution, 93, 150–160. 10.1016/j.ympev.2015.07.024 26256644

[ece35211-bib-0029] Muschick, M. , Indermaur, A. , & Salzburger, W. (2012). Convergent evolution within an adaptive radiation of cichlid fishes. Current Biology, 22, 2362–2368. 10.1016/j.cub.2012.10.048 23159601

[ece35211-bib-0030] Nandamuri, S. P. , Yourick, M. R. , & Carleton, K. L. (2017). Adult plasticity if African cichlids: Rapid change in opsin expression in response to environmental light differences. Molecular Ecology, 26, 6036–6052. 10.1111/mec.14357 28926160 PMC5690868

[ece35211-bib-0031] Pearce, T. (2011). Convergence and parallelism in evolution: A Neo‐Gouldian account. British Journal for the Philosophy of Science, 63, 429–448. 10.1093/bjps/axr046

[ece35211-bib-0032] Plowright, R. C. , & Owen, R. E. (1980). The evolutionary significance of bumble bee color patterns: A mimetic interpretation. Evolution (N.Y.), 34, 622–637. 10.1111/j.1558-5646.1980.tb04002.x 28563986

[ece35211-bib-0033] Rennison, D. J. , Owens, G. L. , Heckman, N. , Schluter, D. , & Veen, T. (2016). Rapid adaptive evolution of colour vision in the threespince stickleback radiation. Proceedings of the Royal Society B, 283, 20160242 10.1098/rspb.2016.0242 27147098 PMC4874711

[ece35211-bib-0034] Ritland, D. , & Brower, L. P. (1991). The viceroy butterfly is not a Batesian mimic. Nature, 350, 497–498. 10.1038/350497a0

[ece35211-bib-0035] Robertson, J. M. , Hoversten, K. , Gründler, M. , Poorten, T. J. , Hews, D. K. , & Rosenblum, E. B. (2011). Colonization of novel White Sands habitat is associated with changes in lizard anti‐predator behaviour. Biological Journal of the Linnean Society, 103, 657–667. 10.1111/j.1095-8312.2011.01644.x

[ece35211-bib-0036] Rosenblum, E. B. (2006). Convergent evolution and divergent selection: Lizards at the White Sands ecotone. American Naturalist, 167, 6389–15. 10.2307/3491243 16475095

[ece35211-bib-0037] Sakai, Y. , Kawamura, S. , & Kawata, M. (2018). Genetic and plastic variation in opsin gene expression, light sensitivity, and female response to visual signals in the guppy. Proceedings of the National Academy of Sciences of the USA, 115, 12247–12252. 10.1073/pnas.1706730115 30420507 PMC6275514

[ece35211-bib-0038] Schluter, D. , Clifford, E. A. , Nemethy, M. , & McKinnon, J. S. (2004). Parallel evolution and inheritance of quantitative traits. American Naturalist, 163, 809–822. 10.1086/383621 15266380

[ece35211-bib-0039] Seehausen, O. (2015). Evolution: Beauty varies with the light. Nature, 521, 34–35. 10.1038/521034a 25951278

[ece35211-bib-0040] Seehausen, O. , Terai, Y. , Magalhaes, I. S. , Carleton, K. L. , Mrosso, H. D. , Miyagi, R. , … Okada, N. (2008). Speciation through sensory drive in cichlid fish. Nature, 455, 620–626. 10.1038/nature07285 18833272

[ece35211-bib-0041] Stevens, M. , Parraga, C. A. , Cuthill, I. C. , Partridge, J. C. , & Troscianko, T. S. (2007). Using digital photography to study animal coloration. Biological Journal of the Linnean Society, 90, 211–237. 10.1111/j.1095-8312.2007.00725.x

[ece35211-bib-0042] Tamura, K. , Stecher, G. , Peterson, D. , Filipski, A. , & Kumar, S. (2013). MEGA6: Molecular evolutionary genetics analysis version 6.0. Molecular Biology and Evolution, 30, 2725–2729. 10.1093/molbev/mst197 24132122 PMC3840312

[ece35211-bib-0043] Vignieri, S. N. , Larson, J. G. , & Hoekstra, H. E. (2010). The selective advantage of crypsis in mice. Evolution (N.Y.), 64, 2153–2158. 10.1111/j.1558-5646.2010.00976.x 20163447

[ece35211-bib-0044] Yokoyama, S. , Yang, H. , & Starmer, W. T. (2008). Molecular basis of spectral tuning in the red‐and green‐sensitive (M/LWS) pigments in vertebrates. Genetics, 179, 2037–2043. 10.1534/genetics.108.090449 18660543 PMC2516078

