# Supplementary material for: Convergent evolution of body color between sympatric freshwater fishes via different visual sensory evolution
Source: Ecol Evol. 2019 Apr 26;9(11):6389–98. doi: 10.1002/ece3.5211 (PMC6580282; doi:10.1002/ece3.5211)
Supplement: Supplementary file 1 [file ECE3-9-6389-s001.pdf]

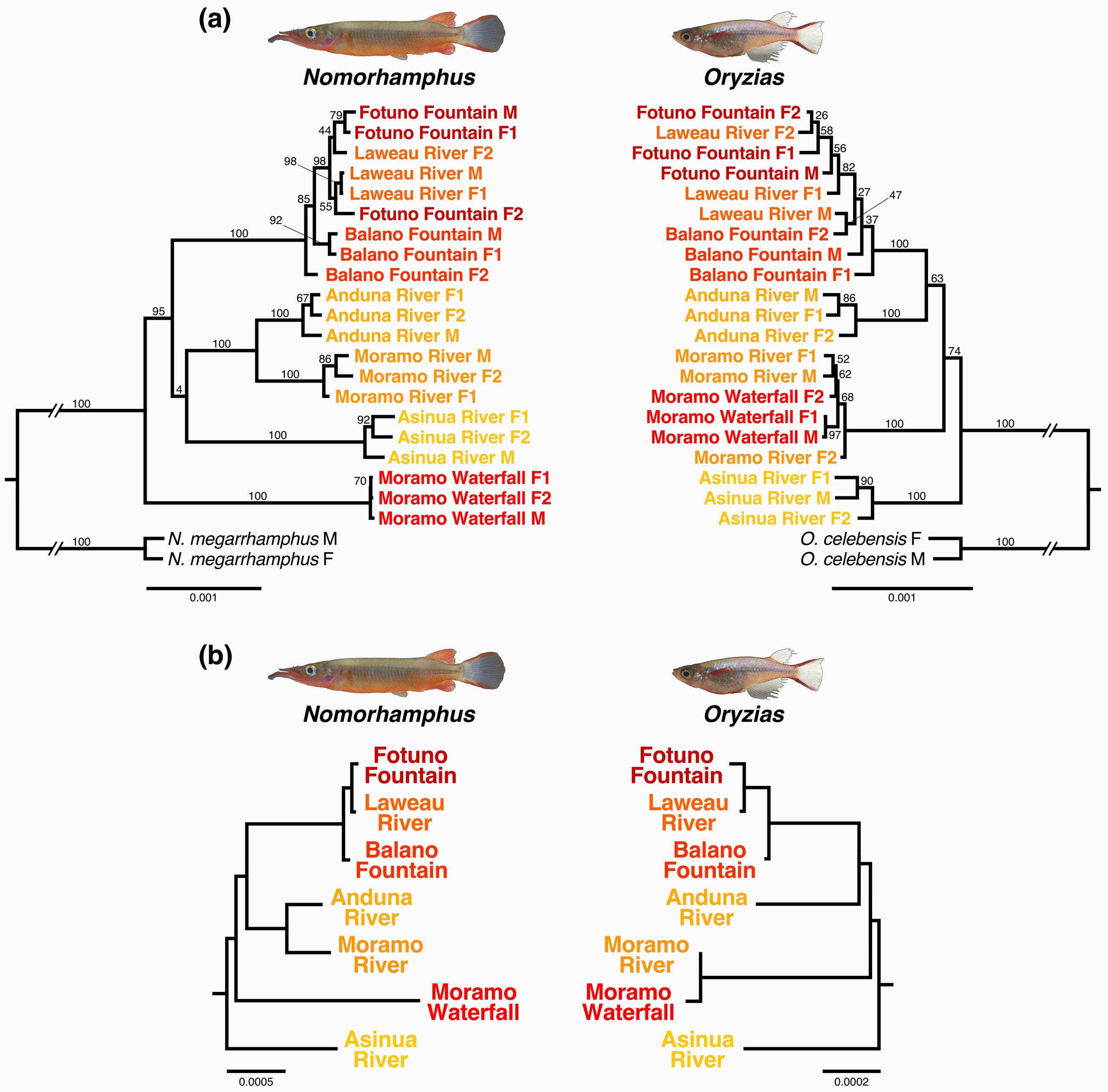

Figure S1 (a) Maximum-likelihood nuclear phylogeny of *Nomorhamphus* and *Oryzias* reconstructed from the concatenating 272 and 148 transcripts, respectively. The numbers on branches show the maximum-likelihood bootstrap values. (b) Neighbor-joining population-averaged tree of *Nomorhamphus* and *Oryzias* based on the d_A values calculated from the 272 and 148 transcripts, respectively.
