# Supplementary material for: Convergent evolution of body color between sympatric freshwater fishes via different visual sensory evolution
Source: Ecol Evol. 2019 Apr 26;9(11):6389–98. doi: 10.1002/ece3.5211 (PMC6580282; doi:10.1002/ece3.5211)
Supplement: Supplementary file 2 [file ECE3-9-6389-s002.pdf]

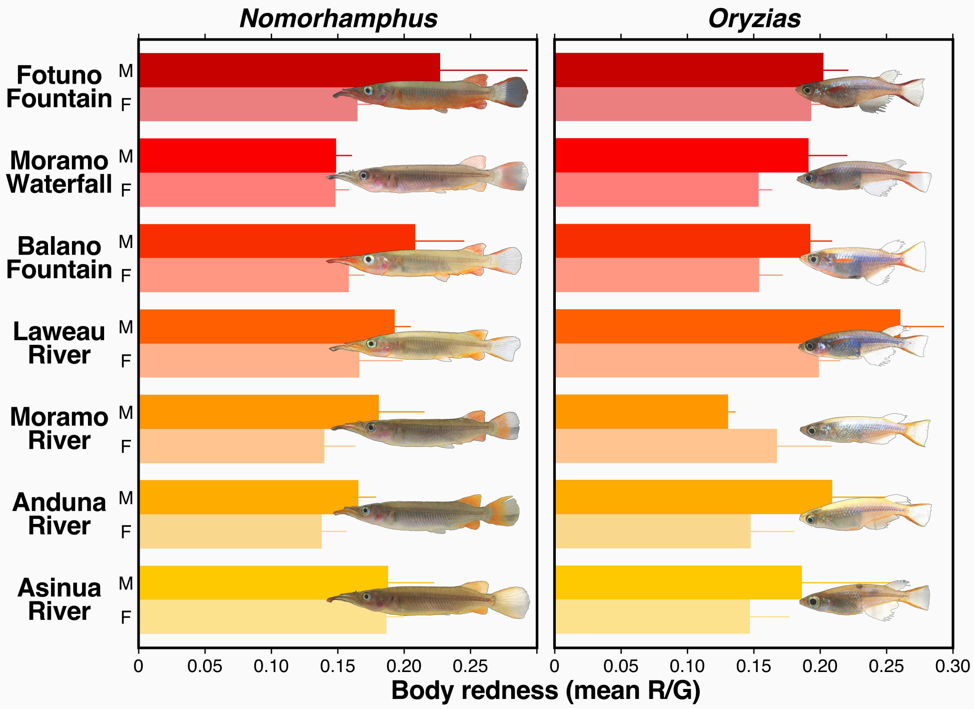

Figure S2 Body redness measured as the mean redness/greenness (R/G) of the wild individuals of *Nomorhamphus* and *Oryzias*.
