# Supplementary figures and images for: Convergent evolution of body color between sympatric freshwater fishes via different visual sensory evolution
Source: Ecol Evol. 2019 Apr 26;9(11):6389–98. doi: 10.1002/ece3.5211 (PMC6580282; doi:10.1002/ece3.5211)

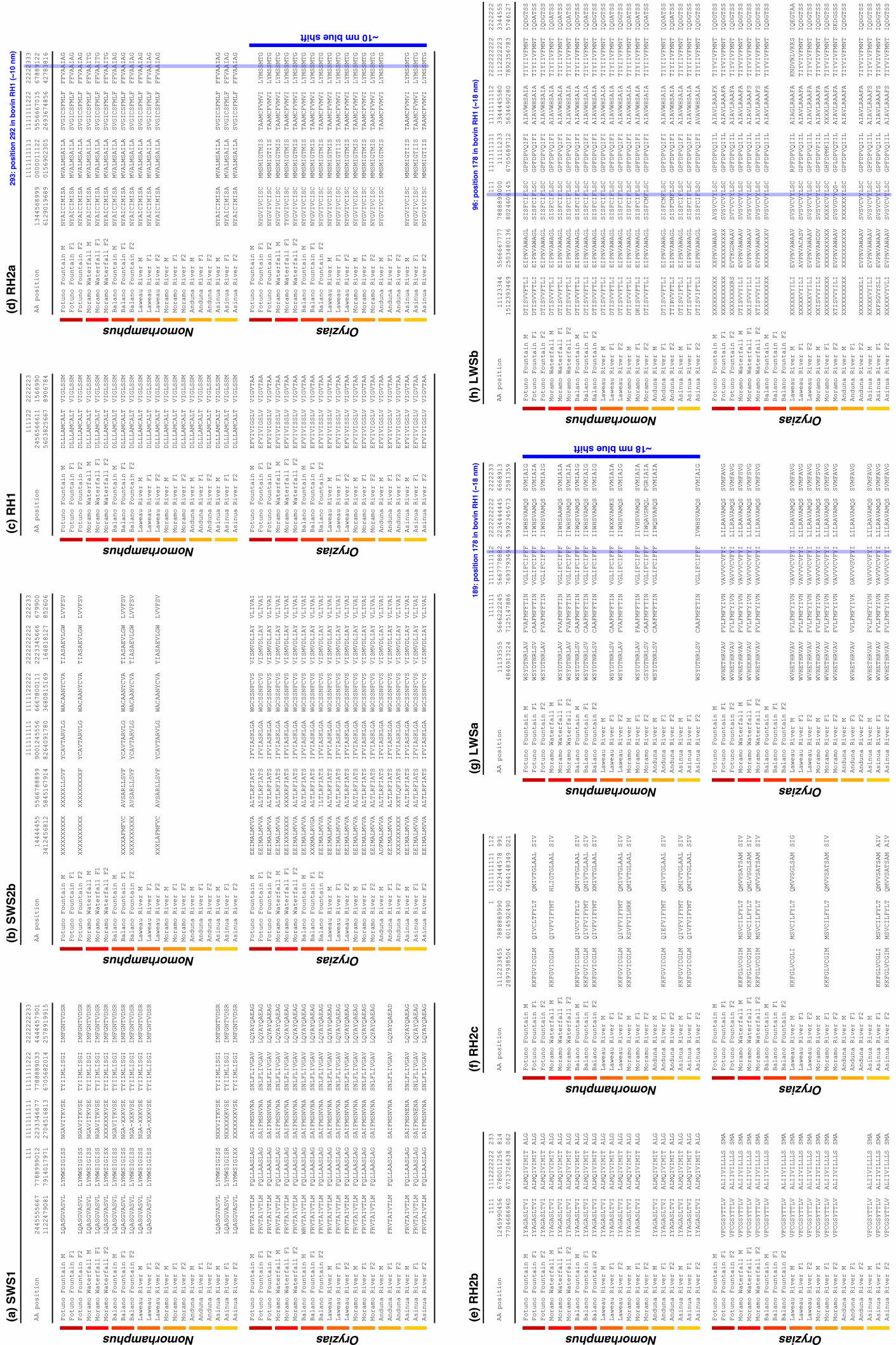

Supplement: Supplementary file 3 [file ECE3-9-6389-s003.pdf]
