# Supplementary material for: Convergent evolution of body color between sympatric freshwater fishes via different visual sensory evolution
Source: Ecol Evol. 2019 Apr 26;9(11):6389–98. doi: 10.1002/ece3.5211 (PMC6580282; doi:10.1002/ece3.5211)
Supplement: Supplementary file 4 [file ECE3-9-6389-s004.pdf]

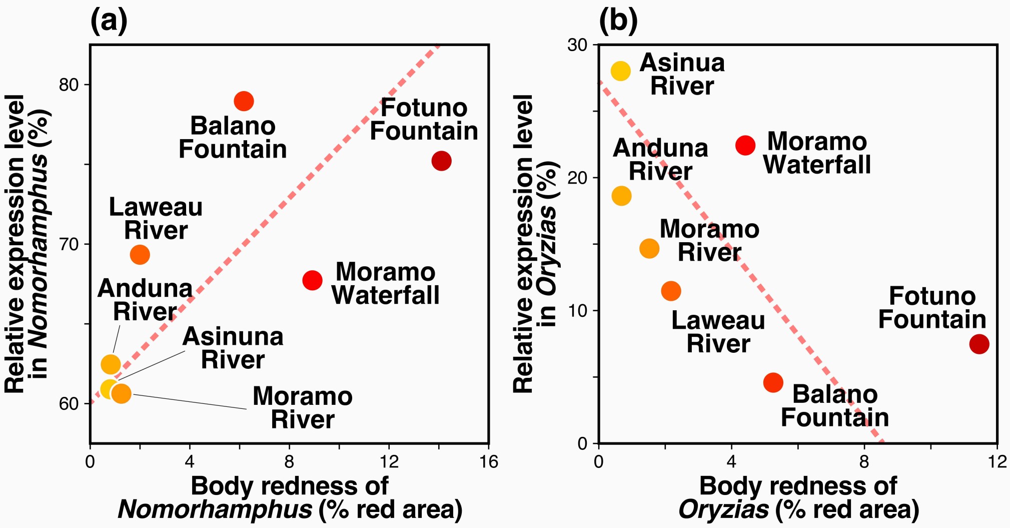

Figure S4 Correlation between the mean ratio of the red areas to the total body area in males and the population-averaged LWS expression level in (a) *Nomorhamphus* and (b) *Oryzias*.
