# Supplementary material for: Convergent evolution of body color between sympatric freshwater fishes via different visual sensory evolution
Source: Ecol Evol. 2019 Apr 26;9(11):6389–98. doi: 10.1002/ece3.5211 (PMC6580282; doi:10.1002/ece3.5211)
Supplement: Supplementary file 5 [file ECE3-9-6389-s005.pdf]

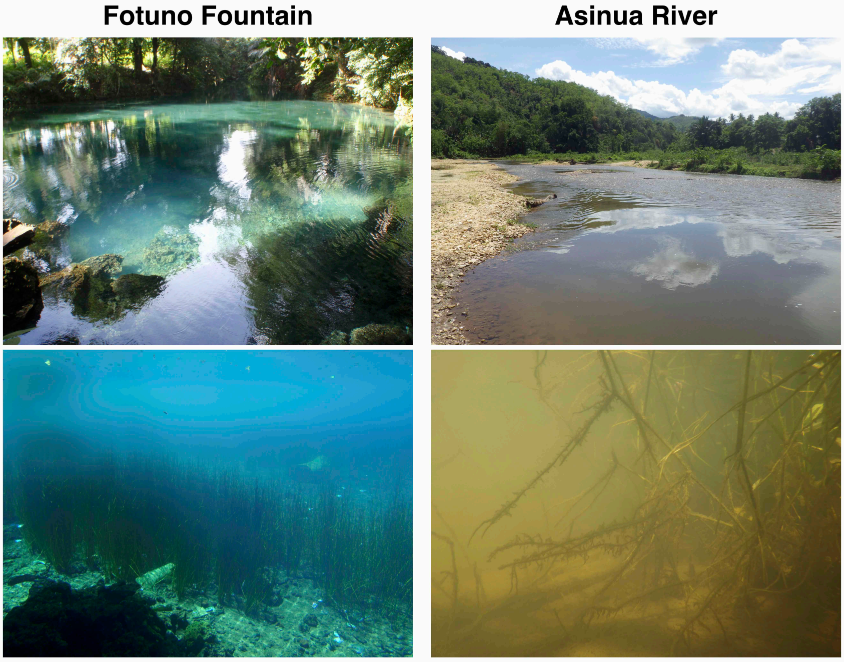

Figure S5 Underwater photography of Fotuno Fountain and Asinua River.
