# Supplementary material for: Convergent evolution of body color between sympatric freshwater fishes via different visual sensory evolution
Source: Ecol Evol. 2019 Apr 26;9(11):6389–98. doi: 10.1002/ece3.5211 (PMC6580282; doi:10.1002/ece3.5211)
Supplement: Supplementary file 9 [file ECE3-9-6389-s009.docx]

**Appendix S1 Testing phenotypic parallelism while adjusting for a potential phylogenetic resemblance**

Phenotypic values of a species are often under the control of natural selection, whilst some endogenous cause or 'phylogenetic constraint' creates the pattern that closely related taxa have more resembling phenotypes. Historically, the latter concept was the rationale on which the cladists of the early days relied on for reconstructing evolutionary histories (Hennig, 1966; Wiley, 1981). For comparative studies on phenotypic adaptation, therefore, the effective sample size is overestimated unless one adjusts for the effect of a potential positive autocorrelation in phenotypic values among taxa, derived from the possible phylogenetic constraint. Numerous solutions have already been proposed (reviewed by Harvey & Pagel, 1991; Garamszegi, 2014), but we implemented the minimum procedure to correct this by using the available molecular distance data.

Relating genetic distance to phenotypic similarity

In this study, we attempted to test whether the observed phenotypic correlation between two taxa simply results from the shared phylogenetic history (null hypothesis) or any other reasons, such as (co)adaptation to sympatric environments. Our approach premises that closer the phylogenetic relationship, stronger is the phenotypic similarity between populations in the same taxon. Equivalently, we postulate that the negative relationship between genetic distance and phenotypic resemblance among populations and the correlation coefficient $\rho_{ij}$ $\left( -1\leq\rho_{ij}\leq1 \right)$ might be mathematically related to the genetic distance $D_{ij} \left( \geq0 \right)$ between the *i*- and *j*-th populations as

$\rho_{ij}=\text{exp}\left( -kD_{ij} \right)$, (1)

where the factor $k \left( >0 \right)$ tunes the strength of their relationship. This equation allows us to convert the genetic distance matrix $\mathbf{D}$ into the phenotype covariance matrix $\boldsymbol{\Sigma}$, of which the element $\left\{ \boldsymbol{\Sigma} \right\}_{ij}$ can be calculated simply as

$\left\{ \boldsymbol{\Sigma} \right\}_{ij}=\sigma^{2}\rho_{ij}$, (2)

where $\sigma^{2}$ is the phenotypic variance among populations. With a mean phenotypic vector $\boldsymbol{\mu=}\left\{ \mu\boldsymbol{,}\mu\boldsymbol{,}\mu\boldsymbol{,}\cdots\boldsymbol{,}\mu\right\}$, we consider that the phenotypic population means follow the multivariate normal distribution $\mathrm{MVN}\left( \boldsymbol{\mu},\boldsymbol{\Sigma} \right)$, in which the parameters $\mu$ and $\sigma^{2}$ could be directly estimated from the observed population means of the phenotypic values. Conversely, a set of random values generated from $\mathrm{MVN}\left( \boldsymbol{\mu},\boldsymbol{\Sigma} \right)$ corresponds to the population-mean phenotypic values that involve the phylogenetic relationship but do not reflect any adaptation to environmental conditions.

A statistic for significance testing

Our interest is whether there is any positive correlation between the phenotypes of two taxa. Accordingly, we define the test statistic as the Pearson correlation coefficient, or

$r=\frac{\sum_{i=1}^{N} \left( x_{i}-\bar{x} \right)\left( y_{i}-\bar{y} \right)}{\sqrt{\sum_{i=1}^{N} \left( x_{i}-\bar{x} \right)^{2}}\sqrt{\sum_{i=1}^{N} \left( y_{i}-\bar{y} \right)^{2}}}$, (3)

where $x_{i}$ and $y_{i}$ are the mean phenotypic values at *i*-th population of taxon A and taxon B, respectively, and *N* is the number of populations (the same between the two taxa). Testing a significant correlation is usually carried out as the statistic $r\sqrt{\left( N-2 \right)/\left( 1-r^{2} \right)}$ follows the Student's *t*-distribution with degrees of freedom $N-2$ under the null hypothesis of no correlation (*r* = 0). However, this routine implicitly assumes that *N* data are statistically independent and does not work for separating phylogenetic constraint-driven and natural selection-driven phenotypic similarities when applied to our data.

Bootstrap distribution of the statistic

Instead of using a Student's *t*-distribution to test the significance of the correlation, we attempt to construct a reference distribution which the statistic follows under the null hypothesis, according to the following parametric bootstrap method:

1. Estimate population mean ($\mu$) and variance ($\sigma^{2}$) from *N* population-mean phenotypic values for taxon A. The vector $\boldsymbol{\mu}$ is prepared by simply lining up *N* $\mu s$.

2. Calculate the phenotype covariance matrix $\boldsymbol{\Sigma}$ from equations (1) and (2) for taxon A.

3. Generate a phenotypic vector $\mathbf{x}$ from $\mathrm{MVN}\left( \boldsymbol{\mu},\boldsymbol{\Sigma} \right)$ for taxon A by randomly choosing a value from the multivariate normal distribution.

4. Do steps 1 to 3 for taxon B to generate a phenotypic vector $\mathbf{y}$.

5. Calculate the statistic *r* by putting $\mathbf{x}$ and $\mathbf{y}$ into equation (3).

6. Repeat this procedure a predetermined number of times (e.g. *B* = 1,000,000).

As large positive values of *r* indicate phenotypic coevolution between the two taxa, obtain the upper-tail *P*-value as

$\text{p}_{\text{upper tail}}=\frac{\text{number of }\text{r}\text{s ≥ }\text{r}_{\text{obs}}}{\text{B}}$, (4)

where $\text{r}_{\text{obs}}$ is the *r* calculated from the original data.

Results

Genetic distances between populations are provided as a matrix of Nei's *d*_A_:

$\mathbf{D}_{x}=0.001\times\left( \begin{matrix} \text{0.0} & \text{2.629} & \text{0.152} & \text{0.061} & \text{1.717} & \text{1.512} & \text{1.899} \\ \text{2.629} & \text{0.0} & \text{2.570} & \text{2.630} & \text{2.110} & \text{2.320} & \text{2.643} \\ \text{0.152} & \text{2.570} & \text{0.0} & \text{0.129} & \text{1.623} & \text{1.414} & \text{1.825} \\ \text{0.061} & \text{2.630} & \text{0.129} & \text{0.0} & \text{1.678} & \text{1.464} & \text{1.875} \\ \text{1.717} & \text{2.110} & \text{1.623} & \text{1.678} & \text{0.0} & \text{0.647} & \text{1.831} \\ \text{1.512} & \text{2.320} & \text{1.414} & \text{1.464} & \text{0.647} & \text{0.0} & \text{1.632} \\ \text{1.899} & \text{2.643} & \text{1.825} & \text{1.875} & \text{1.831} & \text{1.632} & \text{0.0} \end{matrix} \right)$ and

$$\mathbf{D}_{y}=0.001\times\left( \begin{matrix} \text{0.0} & \text{1.203} & \text{0.141} & \text{0.060} & \text{1.121} & \text{0.840} & \text{1.045} \\ \text{1.203} & \text{0.0} & \text{1.046} & \text{1.161} & \text{0.039} & \text{1.070} & \text{1.179} \\ \text{0.141} & \text{1.046} & \text{0.0} & \text{0.110} & \text{0.979} & \text{0.705} & \text{0.859} \\ \text{0.060} & \text{1.161} & \text{0.110} & \text{0.0} & \text{1.078} & \text{0.811} & \text{0.926} \\ \text{1.121} & \text{0.039} & \text{0.979} & \text{1.078} & \text{0.0} & \text{1.006} & \text{1.128} \\ \text{0.840} & \text{1.070} & \text{0.705} & \text{0.811} & \text{1.006} & \text{0.0} & \text{0.976} \\ \text{1.045} & \text{1.179} & \text{0.859} & \text{0.926} & \text{1.128} & \text{0.976} & \text{0.0} \end{matrix} \right)$$

for *Nomorhamphus* and *Oryzias*, respectively. As there is no prior information on the scale of $k$, we computed *P*-values by setting $k=0.1\times max\left( \mathbf{D} \right)$, $\max\left( \mathbf{D} \right)$, and $10\times max\left( \mathbf{D} \right)$, which lead to $\rho$s ranging from $e^{-0.1}$, $e^{-1}$, and $e^{-10}$ to 1.000, respectively. The results are summarized in Tables Appendix. S1.1–S1.3 below.

**Table Appendix. S1.1.** Phenotypic correlations in mean body redness measured as the ratio of the red areas to the total body area between *Nomorhamphus* and *Oryzias* collected from seven populations. The first row contains the original correlation coefficients (*r*s) for males and females. The second to fourth rows indicate *P*-values at different *k*-values, in which a strong phylogenetic effect leads to a greater *P*-value. The fifth row reports the *P*-values when no phylogenetic constraint is assumed (i.e., the non-diagonal elements in **Σ** are all zero). Note that all tests are one-sided.

Male Female

Observed *r* 0.95976 0.64790

$e^{-0.1}\leq\rho\leq$1 0.00193 0.11457

$e^{-1}\leq\rho\leq$1 0.00154 0.10695

$e^{-10}\leq\rho\leq$1 0.00047 0.07249

No adjustment 0.00003 0.06138

**Table Appendix. S1.2.** Phenotypic correlations in mean body redness measured as the mean redness/greenness between *Nomorhamphus* and *Oryzias* collected from seven populations. The first row contains the original correlation coefficients (*r*s) for males and females. The second to fourth rows indicate *P*-values at different *k*-values, in which a strong phylogenetic effect leads to a greater *P*-value. The fifth row reports the *P*-values when no phylogenetic constraint is assumed (i.e., the non-diagonal elements in **Σ** are all zero). Note that all tests are one-sided.

Male Female

Observed *r* 0.14356 0.16389

$e^{-0.1}\leq\rho\leq$1 0.40989 0.39850

$e^{-1}\leq\rho\leq$1 0.40704 0.39501

$e^{-10}\leq\rho\leq$1 0.38908 0.37383

No adjustment 0.27834 0.37042

**Table Appendix. S1.3.** Phenotypic correlations in relative mean expression level of opsin genes between *Nomorhamphus* and *Oryzias* collected from seven populations. The first row contains the original correlation coefficients (*r*s) for each opsin gene. The second to fourth rows indicate *P*-values at different *k*-values, in which a strong phylogenetic effect leads to a greater *P*-value. The fifth row reports the *P*-values when no phylogenetic constraint is assumed (i.e., the non-diagonal elements in **Σ** are all zero). Note that all tests are one-sided.

LWSa+b RH2b+2c RH2a SWS2a+2b SWS1

Observed *r*  -0.79559 -0.33237 0.50946 0.77641 -0.21200

$e^{-0.1}\leq\rho\leq$1 0.04627 0.29454 0.18953 0.05457 0.36805

$e^{-1}\leq\rho\leq$1 0.04168 0.28885 0.18136 0.04911 0.36385

$e^{-10}\leq\rho\leq$1 0.02264 0.25311 0.14191 0.02717 0.33926

No adjustment 0.01489 0.24479 0.13050 0.01911 0.33341

References

Garamszegi, L.Z. 2014. *Modern phylogenetic comparative methods and their application in evolutionary biology.* Springer, Berlin.

Harvey, P.H. & Pagel, M.D. 1991. *The comparative method in evolutionary biology.* Oxford University Press, New York.

Hennig, W. 1966. *Phylogenetic systematics* (tr. D. Dwight Davis and Rainer Zangerl). University of Illinois Press, Illinois.

Wiley, E.O. 1981. *Phylogenetics: the theory and practice of phylogenetic systematics.* Wiley-Interscience, New York.
