# Supplementary material for: Convergent evolution of body color between sympatric freshwater fishes via different visual sensory evolution
Source: Ecol Evol. 2019 Apr 26;9(11):6389–98. doi: 10.1002/ece3.5211 (PMC6580282; doi:10.1002/ece3.5211)
Supplement: Supplementary file 10 [file ECE3-9-6389-s010.docx]

**Supplementary methods**

Phylogenetic reconstruction using the RNA-Seq data

A phylogenetic tree of *Oryzias* in southeast Sulawesi has been reported previously (Mokodongan *et al.*, 2018). Here, we made a phylogenetic tree of *Nomorphamphus* using similar methods. First, we made a reference contigs that can be used for subsequent mapping of short reads. Total RNA was extracted from an eyeball of one female *Nomorphamphus* collected from Fotuno Fountain and preserved in RNAlater® solution (Ambion, Thermo Fisher Scientific, Waltham, USA), using RNeasy Mini Kit (Qiagen, Venlo, The Netherlands). Libraries were prepared using the NEBNext Ultra RNA Library Prep Kit for Illumina (New England BioLabs, Ipswich, USA) and run on the Illumina HiSeq 4000 sequencer with the 2 × 100 bp paired-end mode at Macrogen Japan Corporation, Kyoto, Japan (a total number of reads per sample = 47,934,730). The raw reads were quality-filtered by FaQCs.pl script (Lo & Chain, 2014) and used for *de novo* assembly with Trinity version trinityrnaseq_r20140413 (Grabherr *et al.*, 2011) with default setting. Protein coding sequences were identified using TransDecoder 2.1 (https://github.com/TransDecoder/TransDecoder). The quality of the initial transcriptome assembly was as follows: N50 (weighted median of contig sizes) = 2,109 bp; total size= 112.9 Mb; number of contigs = 109,562. This transcriptome was clustered using CD-HIT-EST (Fu *et al.*, 2012), where contigs with >80% nucleotide sequence identity were considered duplicate genes, splicing variants, and/or alleles, and these duplicate contigs were removed while leaving the longest isoform. The quality of the resulting reference transcriptome was as follows: N50 = 1,476 bp; total size = 30.29 Mb; number of contigs = 28,699.

For phylogenetic analysis of *Nomorhamphus,* fish were collected from seven localities throughout the southeastern arm of Sulawesi and on Muna Island (Fig. 1a): details of sampling sites have been described in Yamahira *et al.* (2016). We collected three individuals (one adult male and two adult females) per site (21 individuals in total), using a beach seine. Two individuals of *Nomorhamphus megarrhamphus* (one male and one female) were also collected from Lake Towuti, central Sulawesi, as outgroups. After euthanasia using MS222, the eyeballs were taken from each individual and preserved in RNAlater® solution.

The transcriptome of these wild individuals (23 individuals in total) were obtained, using the methods described in Mokodongan *et al.* (2018). RNA isolation and library preparation was conducted as described above, and the libraries were run on the Illumina MiSeq sequencer with the 2 × 300 bp paired-end mode using MiSeq Reagent Kit v3 (600 cycles) at the National Institute of Genetics, Mishima, Japan (average read number ± SD per fish = 3,547,162 ± 1,003,754). All raw data were deposited in the DNA Data Bank of Japan (DDBJ) Sequence Read Archive under the accession number DRA006423.

All short reads were trimmed with the FaQCs.pl script and then mapped to the reference contigs, using STAMPY v1.0.23 (Lunter & Goodson, 2011). We excluded transcripts with average per-site coverage of <5 for each individual. We also masked individual sites within a transcript as N, if coverage at that site was <5× or the variant quality score was <40. Heterozygous sites were expressed by the IUPAC ambiguity codes. Nucleotide sequence alignments for each contig were then generated by MAFFT v7.402 with the algorithm L-INS-i (Katoh & Standley, 2013). Only contigs shared among all 23 individuals (including the two *N. megarrhamphus*) were extracted, where 640 contigs of >200 bp with a total alignment length of 0.96 Mbp remained. Thereafter, when two or more contigs were generated from a single reference contig, only the longest one was extracted to avoid linkage disequilibrium between contigs, resulting in the exclusion of five contigs (Table S1). We also excluded three contigs which were revealed to be mitochondrial transcripts by a BLAST search (Table S1). Moreover, we excluded 360 contigs within which one or more populations have a heterozygous site in all of the three individuals, which may represent contigs originated from paralogs (Table S1). Finally, sites containing low-quality bases (masked by “N”) in one or more individuals and the alignment gaps were excluded, resulting in in 272 contigs of >200 bp of a total alignment length of 0.26 Mbp (the number of effective sites of 0.16 Mbp).

After all contigs were concatenated, a maximum likelihood (ML) tree of the 23 individuals of *Nomorhamphus* was estimated using raxmlGUI version 1.31 (Silvestro & Michalak, 2012). In the ML analysis, we set each contig as a separate partition, used the codon-specific GTRCAT models, and assessed the reliability of each tree node by a rapid bootstrap analysis of 1000 replicates. We also reconstructed a coalescent-based population tree, using SVDquartets (Chifman & Kubatko, 2014) implemented in PAUP* 4a (build 163) (http://phylosolutions.com/paup-test/). The analysis was run on the concatenated sequence data set, where we set each contig as a separate partition using the command “charpartition” and assessed the reliability of each tree node by multilocus bootstrapping of 1000 replicates. The concatenated contig alignments and their partition information are archived on Dryad (00000).

Similarly, phylogenetic trees were reconstructed for *Oryzias*, using 456 contigs of >200 bp with a total alignment length of 0.87 Mbp, which had been obtained from RNA-Seq data on 23 individuals, namely the three individuals from each of the seven localities and the two individuals of *O. celebensis* as outgroups, as described in Mokodongan *et al.* (2018). These contigs were filtered by the same criteria above (Table S2), resulting in 148 contigs of >200 bp with a total alignment length of 0.18 Mbp (the number of effective sites of 0.09 Mbp). Using these contigs, a ML tree and a coalescent-based population tree were reconstructed in the same way described above.

A population-averaged phylogenetic tree of the seven populations was also reconstructed separately for *Nomorhamphus* and *Oryzias*, using the number of net nucleotide substitutions per site between two populations (*d*_A_) (Nei & Kumar, 2000), as an index of genetic distance. We calculated a *d*_A_ value of the above transcriptome-wide sequences (the 272 and 148 contigs in *Nomorhamphus* and *Oryzias*, respectively) for each pair among the seven populations, using the command-line version of Arlequin 3.5 (Excoffier *et al.*, 2005). A population-averaged tree was then reconstructed, using the matrix of *d*_A_ values with the neighbor-joining (NJ) method implemented using the program NEIGHBOR of the PHYLIP 3.6 software package (Felsenstein, 2005). The *d*_A_ matrices (Table S3) were also used for the phylogenetic corrections below (see Statistical analyses).

Common-environment experiments

We collected juvenile fish of *Nomorhamphus* from the Fotuno Fountain and Asinua River (four males and four females per site), which had not exhibited secondary sexual characteristics in body redness. Sex of each individual was identified by the morphology of the anal fin; that of the male *Nomorhamphus* functions as an intromittent organ (Meisner, 2001). They were transported alive to the laboratory (Tropical Biosphere Research Center in University of the Ryukyus, Okinawa, Japan). In the laboratory, individuals from the two populations were reared separately but under a common environmental condition (26 °C, 14 L:10 D), and fed daily with frozen brine shrimp (*Artemia franciscana*) and dry food (Kyorin, Hikari Tropical-Fancy Guppy) until they became fully mature (95 days after the laboratory rearing started). Each individual was then measured for the ratio of the red areas to the total body area in the manner described in the main text. One female from Asinua River died before measurement.

We also measured the ratio of the red areas to the total body area of laboratory-reared adult *Oryzias* originating from the Fotuno Fountain and Asinua River. They were maintained for several generations at the World’s Medaka Aquarium, Nagoya Higashiyama Zoological Park, Nagoya, Japan, under a common environment (26 °C, 13 L:11 D), and fed daily with brine shrimp nauplii and dry food. Four mature males and four mature females were randomly chosen from each of the stock populations and used to measure the ratio of the red areas to the total body area as described in the main text.

Measurement of the carotenoid concentrations of the wild-caught individuals

At Fotuno Fountain and Asinua River, five adult males and five adult females of *Nomorhamphus* and three adult males and three adult females of *Oryzias* were collected at each site. They were frozen immediately after collection. In the laboratory, after removing all internal organs from each individual (the head was also removed for *Nomorhamphus*), the weight of the remaining body was measured and acetone extraction was conducted individually until the colour of the body tissue completely disappeared. Following Matsui *et al.* (2002), the solvent was evaporated until dry, and the extract was dissolved in methanol:chloroform (1:3, v/v). The total amount of carotenoids was estimated from the crude extract using a spectrophotometer (U-2900, Hitachi, Japan) at an absorbance of 486 nm with astaxanthin as the standard reference. Carotenoid concentration (nmol/g) was calculated by dividing the total amount of carotenoids by the body weight measured.

**References**

Chifman, J. & Kubatko, L. 2014. Quartet inference from SNP data under the coalescent model. *Bioinformatics* **30:** 3317–3324.

Excoffier, L., Laval, G. & Schneider, S. 2005. An integrated software package for population genetics data analysis. *Evol. Bioinform. Online* **1:** 7.

Felsenstein, J. 2005. PHYLIP (Phylogeny Inference Package) version 3.6. http://evolution.genetics.washington.edu/phylip.html

Fu, L., Niu, B., Zhu, Z., Wu, S. & Li, W. 2012. CD-HIT: accelerated for clustering the next generation sequencing data. *Bioinformatics* **28:** 3150–3152.

Grabherr, M.G., Haas, B.J., Yassour, M., Levin, J.Z., Thompson, D.A., Amit, I. *et al.* 2011. Full-length transcriptome assembly from RNA-seq data without a reference genome. *Nat. Biotechnol.* **29:** 644–652.

Katoh, K. & Standley, D.M. 2013. MAFFT multiple sequence alignment software version 7: improvements in performance and usability. *Mol. Biol. Evol.* **30:** 772–780.

Lo, C.C. & Chain, P.S. 2014. Rapid evaluation and quality control of next generation sequencing data with FaQCs. *BMC Bioinform.* **15:** 366.

Lunter, G. & Goodson, M. 2011. Stampy: a statistical algorithm for sensitive and fast mapping of Illumina sequence reads. *Genome Res*. **21:** 936–939.

Matsui, K., Marunouchi, J. & Nakamura, M. 2002. An ultrastructural and carotenoid analysis of the red ventrum of the Japanese newt, *Cynops pyrrhogaster*. *Pigment Cell Res.* **15:** 265–272.

Meisner, A.D. 2001. Phylogenetic systematics of the viviparous halfbeak genera *Dermogenys* and *Nomorhamphus* (Teleostei: Hemiramphidae: Zenarchopterinae). *Zool. J. Linn. Soc.* **133:** 199–283.

Mokodongan, D.F., Montenegro, J., Mochida, K., Fujimoto, S., Ishikawa, A., Kakioka, R. *et al.* 2018. Phylogenomics reveals habitat-associated body shape divergence in *Oryzias woworae* species group (Teleostei: Adrianichthyidae). *Mol. Phylogenet. Evol.* **118:** 194–203.

Nei, M. & Kumar, S. 2000. *Molecular Evolution and Phylogenetics.* Oxford University Press, New York.

Silvestro, D. & Michalak, I. 2012. raxmlGUI: a graphical front-end for RAxML. *Org. Divers. Evol.* **12:** 335–337.

Yamahira, K., Mochida, K., Fujimoto, S., Mokodongan, D.F., Montenegro, J., Kaito, T. *et al.* 2016. New localities of the *Oryzias woworae* species group (Adrianichthyidae) in Sulawesi Tenggara. *Indonesian J. Ichthyol.* **16:** 125–131.
